# High compliance with the injury prevention exercise programme *Knee Control* is associated with a greater injury preventive effect in male, but not in female, youth floorball players

**DOI:** 10.1007/s00167-021-06644-2

**Published:** 2021-07-02

**Authors:** Ida Åkerlund, Markus Waldén, Sofi Sonesson, Hanna Lindblom, Martin Hägglund

**Affiliations:** 1 Department of Health, Medicine and Caring Sciences, Division of Prevention, Rehabilitation and Community Medicine, Unit of Physiotherapy, Linköping University, S-581 83 Linköping, Sweden; 2 Sport Without Injury ProgrammE (SWIPE), Department of Health, Medicine and Caring Sciences, Linköping University, Linköping, Sweden; 3 Department of Orthopaedics, Hässleholm-Kristianstad Hospitals, Hässleholm, Sweden

**Keywords:** Adherence, Athletic injury, Fidelity, Implementation, Neuromuscular training

## Abstract

**Purpose:**

Evaluate team and player compliance with the *Knee Control* injury prevention exercise programme, study the association between player compliance and injury rates, and compare coach demographics, baseline prevention expectancies, and programme utilisation between teams with high and low compliance.

**Methods:**

Prospective one-season cohort study based on a cluster randomised controlled trial on 301 (107 female) floorball players aged 12–17 years. Floorball exposure and injuries were self-reported weekly by players using the Oslo Sports Trauma Research Center questionnaire. Team and player compliance to *Knee Control* was reported monthly by coaches. Additionally, coaches answered pre- and post-season surveys. Teams were divided into a high (≥ 80%) or low (< 80%) compliance group based on their use of *Knee Control* during the season. Players were divided into three compliance groups based on their average weekly number of *Knee Control* sessions; high (≥ 2 sessions), intermediate (≥ 1 to < 2 sessions), and low dose (< 1 session).

**Results:**

Mean team compliance for the high and low compliance groups were 95% (range 82–100) and 50% (range 13–66), respectively. Mean ± SD weekly *Knee Control* dose in the three player compliance groups were 2.4 ± 0.3, 1.4 ± 0.3, and 0.7 ± 0.3 sessions, respectively. There were no differences in total injury incidence between the player compliance groups, but players in the high-dose group had a 35% lower prevalence of injuries overall [adjusted prevalence rate ratio (PRR) 0.65, 95% CI 0.48–0.89] and 60% lower prevalence of substantial injuries (adjusted PRR 0.40, 95% CI 0.26–0.61) compared with the low-dose group. Male players in the high-dose group had consistently lower injury incidence and prevalence, while no between compliance group differences were seen in female players. There were no differences in sex, years of coaching experience, or baseline prevention expectancies in general between coaches for teams in the high vs. low compliance groups, but teams in the high compliance group had a better utilisation fidelity.

**Conclusion:**

There was a clear dose–response relationship between more frequent *Knee Control* use and lower injury rates in male floorball players, but not in female players. Teams with higher compliance also showed a better utilisation fidelity with the programme.

**Level of evidence:**

Level II.

**Supplementary Information:**

The online version contains supplementary material available at 10.1007/s00167-021-06644-2.

## Introduction

A high number of injuries are reported in many popular youth ball sports [1, 2], despite that several randomised controlled trials (RCTs) have shown substantial injury rate reductions from various injury prevention exercise programmes (IPEPs) [3–5].

The effectiveness of an IPEP depends on both team and player compliance to the programme [6–8]. Compliance may be defined as “an individual conforming to professional recommendations with regard to prescribed dosage, timing and frequency of an intervention” [9]. In a team sport setting, team compliance includes timing and frequency of intervention execution, and depends largely on the motivation and actions of the coach [7]. Player compliance refers to the individual’s intervention dose and is useful to evaluate how compliance influences the effect of the intervention [7].

Many studies show a dose–response relationship with greater injury risk reduction with higher IPEP dose [10–12]. Female youth football players with higher IPEP compliance (mean 1.5 sessions per week) had a 35% lower rate of all injuries compared to players with intermediate compliance (mean 0.7 sessions per week) [7]. Similarly, in another study, female youth football players in the highest compliance tertile (mean 1.4 sessions per week) had 88% lower rate of anterior cruciate ligament (ACL) injury compared to players in the lowest compliance tertile (mean 0.6 sessions per week) [6]. In addition to compliance, having high exercise fidelity (i.e., performing exercises with correct technique) and utilisation fidelity (i.e., which components of an IPEP that are used, when exercises are performed, and the number of sets and repetitions) [13], are also important in achieving a successful implementation of an IPEP [14, 15].

A recent cluster RCT showed that youth floorball players who used the IPEP *Knee Control* had a 45% reduction in acute injury incidence compared with control group players who continued their usual practice [16]. The mean team compliance in that study was high, with *Knee Control* being performed in 84% of training sessions, but varied substantially among teams (range 13–100%). No previous study has evaluated if the level of compliance influences injury rates in youth male or female floorball players. Identifying common factors among teams with high compliance and utilisation fidelity, and the association with programme efficacy, could support future IPEP implementation. The aims of this study on youth floorball players were to evaluate team and player compliance with *Knee Control* and study the association between player compliance and injury rates, and to compare coach demographics, baseline prevention expectancies, and programme utilisation between teams with high and low compliance to *Knee Control*. The hypotheses were that there would be a dose–response relationship between high player compliance to *Knee Control* and lower injury rates, and that highly compliant teams would have more positive prevention expectancies and better programme utilisation than low compliant teams.

## Materials and methods

### Study population and design

Written informed consent was collected from all participating players, and from legal guardians for players below 15 years of age. The study protocol was approved by the Swedish Ethical Review Authority (Dnr 2017/294-31). This prospective cohort study is a sub-analysis of data from a two-armed cluster RCT that evaluated the preventive effect of *Knee Control* (Knäkontroll, SISU Idrottsböcker, Sweden, 2005) on injuries in youth community level floorball players in two districts of Sweden in 2017–2018 [16]. The overall study design, description of *Knee Control*, and main results of the RCT have been reported previously [16].

Briefly, the inclusion criteria for teams in the RCT were: (i) male and female players aged 12–17 years, (ii) had not used any IPEP regularly in the last year, and (iii) had ≥ 2 scheduled team training sessions per week. The exclusion criteria were: (i) mixed-age teams with most of their players being outside the age range 12–17 years, and (ii) individual players within included teams who were outside the age range. In total, 31 teams (8 female) with 301 players (107 female) in 17 clubs were included in the intervention arm of the RCT and included in this sub-analysis.

### Intervention programme and delivery methods

The intervention consisted of a standardised 5-min running warm-up, followed by *Knee Control* with three sets of 8–15 repetitions for each exercise (programme duration 10–15 min). *Knee Control* consists of six principal exercises (one-legged knee squat, pelvic lift, two-legged knee squat, the bench, the lunge, and jump/landing technique). Each exercise has three or four steps (A–C/D) of progression with increasing difficulty and one partner exercise for variation (online additional file 1). The coaches were instructed to use the running warm-up and *Knee Control* before every training session throughout the 26-week season, starting at the easiest level (A) and progress individually as players’ strength, balance, neuromuscular control, and technique improved.

The intervention group coaches plus 1–2 players per team were invited to a 3-h implementation workshop at the beginning of the floorball season (September 2017). Coaches and represented players received practical instructions about the correct execution of the warm-up running exercises and *Knee Control* exercises. The programme was also made available to coaches in video format, and written instructions with explanatory text and pictures were provided.

### Definitions and data collection

Injury and compliance definitions are presented in Table 1. The injury definition involves both acute and gradual onset injuries. Players answered a weekly web-based survey with questions about match and training exposure and occurrence of any injury in the past week. If an injury was reported, the player answered the four questions of the Oslo Sports Trauma Research Center questionnaire to evaluate its impact on sports participation, training volume, performance, and pain [17].

**Table 1:** Injury and compliance definitions

Injury	Any physical complaint sustained by a player that results from floorball training or match, irrespective of the need for medical attention or time-loss from floorball activities [25], i.e., when a player recorded any option other than 0 on the modified Oslo Sports Trauma Research Center (OSTRC) questionnaire [26].
Acute injury	Injury that occurred suddenly and was associated with a specific, identifiable event [25].
Gradual onset injury	Injury caused by repeated microtrauma without a single, identifiable event responsible for the injury [25].
Substantial injury	Injury having a moderate or major effect on reduction in training volume or performance, or inability to participate in floorball according to player registration in the OSTRC questionnaire [26].
Time loss injury	Injury that caused absence from floorball training or match play [25].
Injury event	Any new injury or recurrent injury occurring after the player had reported at least 1 week of full floorball participation without any health problems between the index injury and the subsequent injury. Multiple consecutive weeks of the same reported health problem (e.g., several weeks of time-loss from play or several weeks affected training volume, performance, participation, or pain) were considered as the same injury event in injury incidence calculations.
Team compliance	The proportion of all registered team training sessions and matches where the coach reported use of *Knee Control* and the running warm-up [7]. Reported as a season proportion.
Player compliance	Individual player dose, i.e., the number of training sessions where *Knee Control* was used and where the player attended the training session [7]. Reported as a weekly average.
Utilisation fidelity	Exercise selection, and timing of *Knee Control* (before, during, or after the training session), and the number of sets and repetitions of *Knee Control* as reported by coaches [13].

Coaches documented each scheduled team training session and match and the individual player participation in these activities on a standard attendance form. For each training session, the coach also documented whether the team had completed *Knee Control* (yes/no), allowing for calculation of both team and individual player compliance to *Knee Control*. A pre-season survey with 11 questions relating to coach demographics and injury prevention expectancies, based on a previously used questionnaire [18], and adapted to a floorball context (online additional file 2) was sent out to all coaches who were registered in the team. A post-season coach survey was also conducted after completing the prospective injury registration with 22 questions about, for example, utilisation fidelity of *Knee Control* (online additional file 2).

### Statistical analysis

**Fig. 1:**
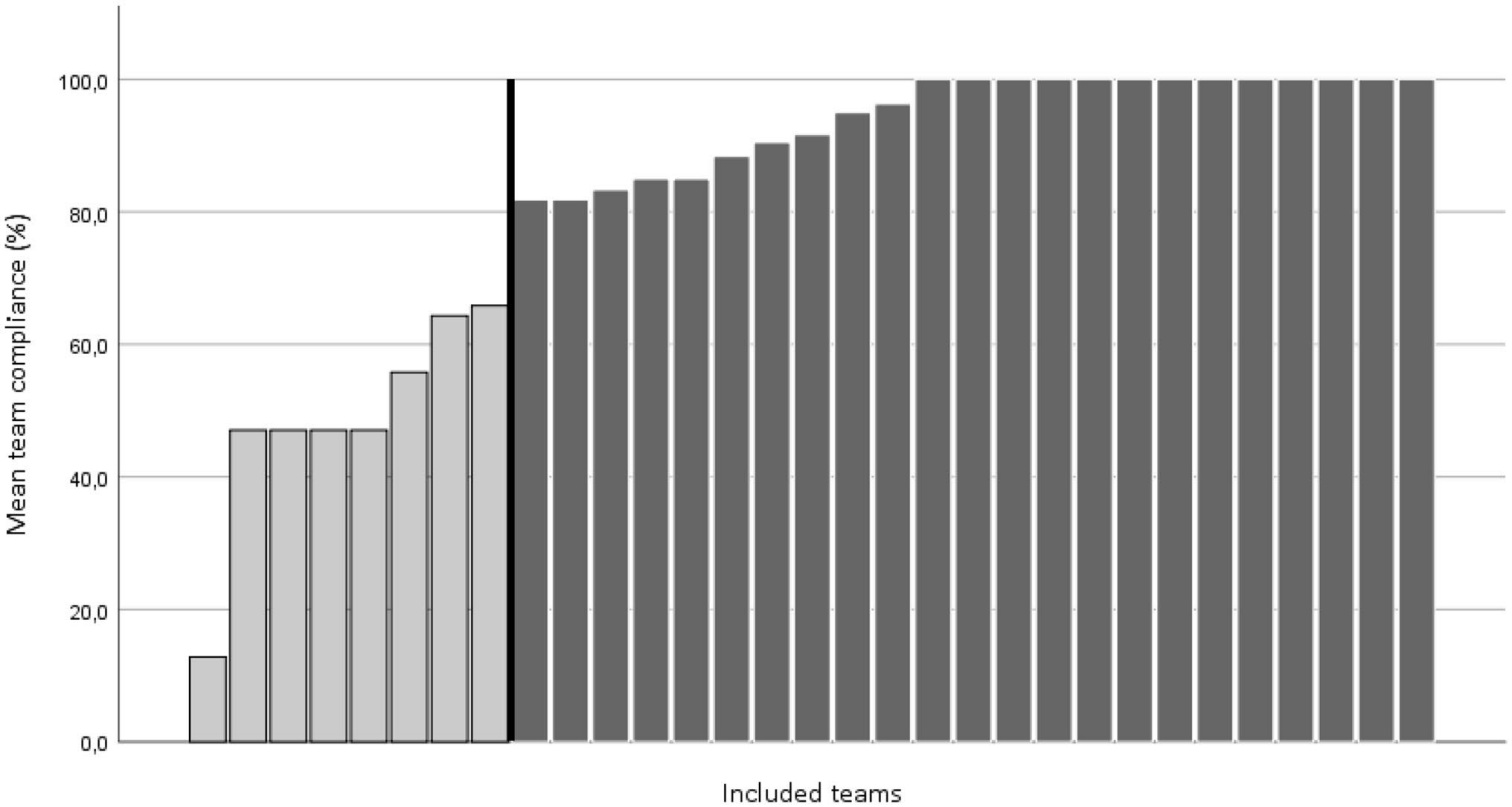
Distribution and cut-off for team compliance (the proportion of all registered team training sessions where the coach reported use of *Knee Control*, mean season proportion) among low compliance (light grey bars) and high compliance teams (dark grey bars)

Teams were divided by a skewed dichotomization (Fig. 1) into two compliance groups based on their reported use of *Knee Control* over the whole season: high compliance (used *Knee Control* for 80–100% of training sessions) and low compliance (< 80% use). Players were allocated to three expedient compliance groups based on their average weekly use of *Knee Control*: high dose (≥ 2 *Knee Control* sessions per week), intermediate dose (≥ 1 to < 2 *Knee Control* sessions per week), and low dose (< 1 *Knee Control* session per week). This pragmatic categorization was used to increase the feasibility of implementing the results.

Injury incidence is expressed as the number of unique injury events per 1000 h of floorball play. Weekly injury prevalence is expressed as the number of weekly reports where a player reported a floorball injury (new or ongoing) divided by the total number of eligible player reports each week. No imputation was made for missing data. Incidence for all, acute onset and time-loss injuries, and prevalence for all, gradual onset and substantial injuries are reported. Descriptive statistics were used for demographic data and are presented as means and standard deviations (SDs) and/or range, medians and interquartile ranges (IQRs), or numbers and proportions (%). Generalized estimating equations (GEEs) were used to analyse the weekly data and we applied a Poisson distribution with a log link function, and model-based standard error estimation, to calculate a rate ratio (RR) and corresponding 95% confidence interval (CI) for injury incidence and prevalence differences between compliance groups. The low-dose group was used as reference group in the analyses. The overall GEE analyses were adjusted for sex, and also reported by sex separately, and due to a skewed age distribution between player compliance groups, also adjusted for age. All analyses were performed using SPSS statistical software for Windows (v26; IBM, New York), and a *p* value < 0.05 was considered to be significant. A sample size calculation was made for the main effect between the intervention and control group in the cluster RCT [16], while no a priori sample size calculation was done for this sub-analysis of the intervention group.

## Results

### Team and player compliance

Mean team season compliance for the high and low compliance groups was 95% (range 82–100%, teams *n* = 23) and 50% (range 13–66%, teams *n* = 8), respectively (Fig. 1). Mean weekly dose of *Knee Control* for players was 1.45 ± 1.02 sessions per week. Player demographics, floorball exposure, and player compliance for the three player compliance groups are presented in Table 2.

**Table 2:** Player demographics, floorball exposure and compliance stratified into low, intermediate, and high dose of *Knee Control*

	Age, years mean (SD)	Menarche, yes N (%)	Exposure (training and match), h/week mean (SD)	*Knee Control* dose, sessions per week mean (SD, range)
Low dose				
Total (*n* = 64)	13.6 (0.8)		3.9 (1.4)	0.7 (0.3, 0–1.0)
Female (*n* = 23)	14.0 (0.9)	20 (95)^a^	3.7 (0.7)	0.4 (0.3, 0–1.0)
Male (*n* = 41)	13.4 (0.7)		3.9 (1.6)	0.8 (0.2, 0.2–1.0)
Intermediate dose				
Total (*n* = 185)	13.6 (1.1)		4.0 (1.4)	1.4 (0.3, 1.0–2.0)
Female (*n* = 65)	13.5 (1.3)	37 (57)	3.5 (1.1)	1.3 (0.3, 1.0–2.0)
Male (*n* = 120)	13.6 (0.9)		4.2 (1.6)	1.5 (0.3, 1.0–1.9)
High dose				
Total (*n* = 52)	13.5 (1.5)		4.9 (1.7)	2.4 (0.3, 2.0–3.0)
Female (*n* = 18)	14.7 (1.5)	18 (100)	5.0 (1.3)	2.4 (0.3, 2.0–2.8)
Male (*n* = 34)	12.9 (1.1)		4.8 (1.9)	2.4 (0.3, 2.0–3.0)

Low dose: < 1 *Knee Control* session per week, intermediate dose: ≥ 1 to < 2 *Knee Control* sessions per week, high dose: ≥ 2 *Knee Control* sessions per week

^a^Missing for two girls

### Player compliance and injury rates

There were 197 injuries reported in 135 (45%) players. Forty percent of injuries resulted in time-loss. The lower limb, especially the knee joint, was the most common injury location in all compliance groups. Injury panorama divided into acute and gradual onset is shown in Table 3.

**Table 3:** Number of injuries among players stratified into low, intermediate, and high dose of *Knee Control*

	Low dose		Intermediate dose		High dose	
	Acute	Gradual onset	Acute	Gradual onset	Acute	Gradual onset
All injuries, *n*	8	25	31	93	13	27
Time loss, *n* (%)	7 (88)	9 (36)	16 (52)	35 (38)	5 (38)	7 (26)
New injury, *n* (%)	7 (88)	7 (28)	23 (74)	35 (38)	10 (77)	5 (19)
Reinjury, *n* (%)	1 (12)	18 (72)	8 (26)	58 (62)	3 (23)	22 (81)
Injury location, *n* (%)						
Lower limbs	4 (50)	19 (76)	25 (81)	76 (82)	11 (85)	20 (74)
Hip/groin	1 (13)	1 (4)	3 (10)	3 (3)	2 (15)	3 (11)
Thigh	1 (13)	0	8 (26)	4 (4)	2 (15)	0
Knee	1 (13)	13 (52)	7 (23)	53 (57)	1 (8)	12 (44)
Lower leg/Achilles tendon	0	2 (8)	1 (3)	8 (9)	2 (15)	0
Ankle	1 (13)	1 (4)	4 (13)	1 (1)	3 (23)	0
Foot/toe	0	2 (8)	2 (6)	7 (8)	1 (8)	5 (19)
Trunk	2 (25)	0	2 (6)	9 (10)	1 (8)	2 (7)
Upper limbs	1 (13)	2 (8)	3 (10)	3 (3)	0	3 (11)
Head and neck	1 (13)	0	1 (3)	1 (1)	1 (8)	2 (7)
Other	0	0	0	1 (1)	0	0

Low dose (*n* = 64): < 1 *Knee Control* session per week, intermediate dose (*n* = 185): ≥ 1 to < 2 *Knee Control* sessions per week, high dose (*n* = 52): ≥ 2 *Knee Control* sessions per week

There were no statistically significant differences in total injury incidence between the compliance groups (Table 4). The sex-specific analyses indicated a significant dose–response relationship between higher *Knee Control* use and a greater injury rate reduction in males, while there were no apparent associations in female players.

**Table 4:** Injury incidence rates among players stratified into low, intermediate, and high dose of *Knee Control*

	Number of injuries	Incidence (95% CI)	Age-adjusted incidence (95% CI)	Rate ratio vs. low-dose group (95% CI)^b^	*p* value
All injuries^a^					
Low dose	33	15.3 (10.9–21.6)	15.2 (10.8–21.5)	–	
Intermediate dose	124	11.7 (9.8–13.9)	11.7 (10.0–13.9)	0.77 (0.52–1.13)	(n.s)
High dose	40	11.3 (8.3–15.4)	11.2 (8.2–15.3)	0.73 (0.46–1.17)	(n.s)
Time loss injuries^a^					
Low dose	11	7.6 (4.6–12.4)	7.6 (4.6–12.4)	–	
Intermediate dose	30	4.8 (3.6–6.3)	4.8 (3.6–6.3)	0.63 (0.36–1.12)	(n.s)
High dose	6	3.4 (1.9–6.0)	3.4 (1.9–6.0)	0.45 (0.21–0.95)	0.036
Acute injuries^a^					
Low dose	8	3.7 (1.8–7.4)	3.6 (1.8–7.3)	–	
Intermediate dose	31	2.9 (2.1–4.2)	2.9 (2.0–4.1)	0.80 (0.37–1.76)	(n.s)
High dose	13	3.7 (2.1–6.3)	3.6 (2.0–6.2)	0.98 (0.41–2.38)	(n.s)
All injuries male players					
Low dose	21	19.1 (12.4–29.2)	18.9 (12.3–29.1)	–	
Intermediate dose	80	11.5 (9.2–14.3)	11.6 (9.3–14.4)	0.61 (0.38–1.00)	0.047
High dose	20	9.2 (5.9–14.2)	8.9 (5.6–14.1)	0.47 (0.25–0.88)	0.018
Time loss injuries male players					
Low dose	6	10.0 (5.5–18.0)	10.0 (5.5–18.0)	–	
Intermediate dose	21	4.9 (3.5–6.8)	4.9 (3.5–6.9)	0.50 (0.25–0.99)	0.046
High dose	2	2.8 (1.2–6.1)	2.7 (1.2–6.2)	0.27 (0.10–0.74)	0.011
Acute injuries male players					
Low dose	5	4.5 (1.9–10.9)	4.6 (1.9–11.1)	–	
Intermediate dose	22	3.2 (2.1–4.8)	3.1 (2.0–4.8)	0.67 (0.25–1.80)	(n.s)
High dose	4	1.8 (0.7–4.9)	1.9 (0.7–5.2)	0.42 (0.11–1.57)	(n.s)
All injuries female players					
Low dose	12	11.6 (6.6–20.4)	11.0 (6.2–20.0)	–	
Intermediate dose	44	12.1 (9.0–16.2)	12.4 (9.2–16.8)	1.13 (0.59–2.17)	(n.s)
High dose	20	14.7 (9.5–22.8)	13.0 (7.8–21.6)	1.18 (0.56–2.45)	(n.s)
Time loss injuries female players					
Low dose	5	4.8 (2.0–11.6)	4.9 (2.0–11.8)	–	
Intermediate dose	9	4.7 (2.9–7.5)	4.6 (2.8–7.6)	0.95 (0.34–2.68)	(n.s)
High dose	4	4.4 (2.0–9.8)	4.5 (1.9–10.9)	0.93 (0.28–3.11)	(n.s)
Acute injuries female players					
Low dose	3	2.9 (0.9–9.0)	3.0 (1.0–9.3)	–	
Intermediate dose	9	2.5 (1.3–4.8)	2.4 (1.2–4.7)	0.81 (0.21–3.10)	(n.s)
High dose	9	6.6 (3.4–12.7)	7.1 (3.4–15.2)	2.40 (0.64–9.03)	(n.s)

Low dose (*n* = 64): < 1 *Knee Control* session per week, intermediate dose (*n* = 185): ≥ 1 to < 2 *Knee Control* sessions per week, high dose (*n* = 52): ≥ 2 *Knee Control* sessions per week

^a^Model adjusted for sex

^b^Calculated from age-adjusted injury incidence

Players in the high-dose group had 35% and 60% lower weekly prevalence of any floorball injury and substantial injury, respectively, compared with the low-dose group. Consistently, males in the intermediate- and high-dose groups had lower prevalence rates for all injuries, substantial injuries, and gradual onset injury compared with the low-dose group, while no differences between compliance groups were seen for female players (Table 5).

**Table 5:** Weekly prevalence of floorball injuries among players stratified into low, intermediate, and high dose of *Knee Control*

	Prevalence (95% CI)	Age-adjusted prevalence (95% CI)	Rate ratio vs. low-dose group (95% CI)^b^	*p* value
All injuries^a^				
Low dose	14.8 (12.0–18.3)	14.5 (11.7–17.9)	–	
Intermediate dose	12.0 (10.8–13.3)	12.0 (10.8–13.4)	0.83 (0.66–1.05)	(n.s)
High dose	9.7 (7.7–12.2)	9.4 (7.5–11.9)	0.65 (0.48–0.89)	0.006
Substantial injuries^a^				
Low dose	10.5 (8.2–13.5)	10.0 (7.8–12.9)	–	
Intermediate dose	7.0 (6.1–8.0)	6.9 (6.0–8.0)	0.69 (0.52–0.92)	0.011
High dose	4.3 (3.1–6.1)	4.0 (2.8–5.7)	0.40 (0.26–0.61)	< 0.001
Gradual onset injuries^a^				
Low dose	9.6 (7.4–12.4)	9.4 (7.3–12.3)	–	
Intermediate dose	9.4 (8.4–10.7)	9.5 (8.4–10.7)	1.00 (0.75–1.33)	(n.s)
High dose	6.8 (5.2–8.9)	6.8 (5.1–8.9)	0.71 (0.49–1.04)	(n.s)
All injuries male players				
Low dose	16.3 (12.3–21.7)	16.5 (12.4–21.9)	–	
Intermediate dose	10.9 (9.5–12.6)	10.8 (9.4–12.5)	0.66 (0.48–0.90)	0.010
High dose	6.7 (4.7–9.4)	6.9 (4.8–9.8)	0.42 (0.27–0.66)	< 0.001
Substantial injuries male players				
Low dose	11.9 (8.5–16.6)	12.3 (8.8–17.1)	–	
Intermediate dose	6.6 (5.5–7.9)	6.0 (4.9–7.3)	0.49 (0.33–0.72)	< 0.001
High dose	1.5 (0.7–3.1)	1.7 (0.8–3.5)	0.14 (0.06–0.31)	< 0.001
Gradual onset injuries male players				
Low dose	11.9 (8.5–16.6)	11.8 (8.4–16.4)	–	
Intermediate dose	8.1 (6.9–9.5)	8.2 (6.9–9.6)	0.69 (0.48–1.01)	(n.s)
High dose	5.8 (4.0–8.4)	5.6 (3.8–8.2)	0.47 (0.29–0.78)	0.004
All injuries female players				
Low dose	14.8 (10.9–20.0)	13.8 (10.1–18.8)	–	
Intermediate dose	14.2 (12.1–16.7)	14.9 (12.7–17.5)	1.08 (0.76–1.53)	(n.s)
High dose	15.4 (11.4–20.8)	12.8 (9.1–17.9)	0.93 (0.60–1.44)	(n.s)
Substantial injuries female players				
Low dose	10.2 (7.1–14.7)	9.7 (6.7–14.1)	–	
Intermediate dose	8.0 (6.4–10.0)	8.3 (6.6–10.3)	0.85 (0.55–1.31)	(n.s)
High dose	9.5 (6.5–14.0)	8.4 (5.5–12.9)	0.86 (0.50–1.49)	(n.s)
Gradual onset injuries female players				
Low dose	8.1 (5.4–12.2)	7.5 (4.9–11.3)	–	
Intermediate dose	12.1 (10.2–14.4)	12.8 (10.7–15.2)	1.71 (1.09–2.68)	0.021
High dose	8.8 (5.9–13.1)	7.1 (4.6–11.0)	0.95 (0.53–1.70)	(n.s)

Low dose (*n* = 64): < 1 *Knee Control* session per week, intermediate dose (*n* = 185): ≥ 1 to < 2 *Knee Control* sessions per week, high dose (*n* = 52): ≥ 2 *Knee Control* sessions per week. Substantial injuries were those that lead to moderate or severe reductions in training volume or performance, or inability to participate in floorball

^a^Model adjusted for sex

^b^Calculated from age-adjusted injury prevalence

### High vs. low compliance teams

Coaches in the two team compliance groups were similar regarding demographics and baseline prevention expectancies except for perceived performance effects (Table 6). Coaches in the high compliance group believed that regular use of an IPEP would increase a player’s performance, while coaches in the low compliance group stated that performance would decrease.

**Table 6:** Team and coach demographics, prevention expectancies, and utilisation fidelity stratified into low and high team compliance

	Low compliance teams N = 6 coaches	High compliance teams *N* = 42 coaches
Number of teams (female)	8 (1)	23 (7)
Training sessions per week, mean (SD, range)	2.1 (0.6, 1.8–3.4)	2.1 (0.8, 1.4–3.1)
Matches per week, mean (SD, range)^a^	1.0 (1.1, 0.4–3.2)	0.9 (0.9, 0.6–1.6)
Coach demographics		
Coach sex male/female, *n*	5/1	36/6
Coaching experience years, median (IQR, range)^b^	7 (2, 5–7)	5 (4, 1–22)
Pre-season survey: prevention expectancies		
What is your opinion about the overall injury risk in floorball? median (IQR) (1 extremely low–7 extremely high)	5 (2)	4 (2)
In general, how preventable do you think floorball injuries are? median (IQR) (1 extremely not preventable–7 extremely preventable)	6 (1)	6 (1)
My knowledge about preventing injuries in floorball is… median (IQR) (1 extremely poor–7 extremely good)	4 (2)	4 (2)
In your opinion, what would happen to a floorball player’s overall risk of injury if he/she participated in injury prevention training? median (IQR) (1 increase extremely–7 decrease extremely)	4.5 (4)	5.5 (4)
What do you think would happen to a floorball player’s performance if he/she did injury prevention training regularly? median (IQR) (1 decrease extremely–7 increase extremely)	3 (3)	5.5 (1)
Post-season survey: *Knee Control* utilisation fidelity^c^		
* Knee Control* dose, sessions per week mean (SD, range)	1.1 (0.8, 0.2–1.6)	2.0 (0.8, 1.2–3.0)
* Knee Control* use, min/training session mean (SD, range)	14 (8, 5–25)	17 (5, 10–25)
* Knee Control* number of sets, median (IQR)^d^	2 (0)	2 (2)
* Knee Control* use, min/week	15	34
Timing of *Knee Control* use^d^		
Before training session, %	0	25
Beginning of training session, %	100	75
During training session, %	0	0
After training session, %	0	0
Use of all six *Knee Control* exercises		
Mostly/always, %	50	91
Never/rarely, %	50	9
* Knee Control* progression level, median (IQR, range)^e,f^	1 (1, 1–3)	2 (1, 1–4)

Low compliance < 80%, high compliance 80–100%

^a^Missing for one team in high compliance group

^b^Missing for one coach in low compliance group and one coach in high compliance group

^c^Only one response per team included. Missing data for one team in the low compliance group

^d^Missing for one team in the high compliance group

^e^Missing for two coaches in high compliance group

^f^Progression levels A (easiest) to D (most advanced) numbered from 1 to 4

More teams in the high compliance group than in the low compliance group reported use of all six exercises of *Knee Control* (as per instructions) on most training sessions (Table 6 and online additional file 3). Teams in the high compliance group reported using varied progression levels during different training sessions and had progressed to using level B and C exercises, while teams in the low compliance group used mainly level A (easiest) exercises.

## Discussion

The most important findings of the present study were that a higher *Knee Control* weekly dose was associated with lower injury incidence of time-loss and any floorball injuries and injury prevalence of substantial, gradual onset and any floorball injuries for male youth floorball players, while no such association was seen in female players. Three out of four teams used *Knee Control* in 80% or more of their training sessions over the season and were regarded as highly compliant. In addition to a higher frequency of *Knee Control* use, coaches in the high team compliance group had adopted the full programme to a higher degree, i.e., they more often used all six exercises of the IPEP, and had progressed the exercises over time and incorporated more challenging exercise options.

### Greater preventive effect with a higher dose in male players

There was a clear dose–response relationship between high *Knee Control* dose and lower injury incidence and injury prevalence in male players. Male players in the high-dose group had 53% lower incidence and 58% lower weekly prevalence of floorball injury overall, compared with players in the low-dose group. They also had a lower injury burden, with 73% lower incidence of time-loss injuries and 86% lower weekly prevalence of substantial injuries, compared with players in the low-dose group. These results align with the previous research on male and female players in different team ball sports [6, 7, 10–12, 19]. Male players in the intermediate-dose group (mean 1.5 ± 0.3 sessions per week) also had a greater injury preventive effect compared with the low-dose group, but less pronounced compared with the high-dose group. In summary, male players who had at least one *Knee Control* session per week showed an injury preventive effect, but players with two or more *Knee Control* sessions per week had the greatest preventive effect. This aligns with a recent meta-analysis that showed the highest injury reductions when an IPEP was performed two to three times per week and with a weekly volume of 30–60 min [11]. Importantly, given an average player training attendance of 71% in youth sports [6], coaches should aim to use *Knee Control* on minimum three training sessions per week to reach an average player dose of two weekly *Knee Control* sessions.

### No dose–response relationship in female players

In contrast to the dose–response relationship in male players, there were no differences in injury rates between the player compliance groups in female players. This contradicts the previous findings in youth female athletes [2, 11, 12, 20] and it is unlikely that injury reduction effects from our IPEP would be less evident in female floorball players. A small number of female players and few injuries made the analyses less robust, and the risk of sampling bias and type-2 error must be acknowledged. Females in the high-dose IPEP group had the highest floorball exposure, and general overload in this group could be one explanation. In the main RCT, we showed a possible adverse intervention effect on gradual onset knee injuries for females with two times higher prevalence rate in the intervention group vs. the control group [16]. For developing youth players, it is important to find a balance between sport exposure, injury prevention training, and recovery. If the players already have a high training load, addition of an IPEP could result in a too high total workload and risk of gradual onset injury [21]. It is important to help coaches adjust the total workload and implement the IPEP gradually, e.g., start with a lower number of sets or exercises and then progress to the recommended dose. The risk of gradual onset injuries also increases during the pubertal growth spurt, and girls reach their peak height and body mass at approximately age 15 [21], which coincides with the female players’ mean age in the high-dose group in this study. As a general consideration, it could be advantageous to introduce an IPEP at an early age before the growth spurt, so that players may benefit from the IPEP preventive effects early on and are accustomed to injury prevention exercises at the onset of puberty. Injury risk factors may differ between male and female players and need to be explored in future studies. To get more information about the injury preventive effect, and how and why it differs between male and female players, it is of interest to study the effect mechanisms of *Knee Control*.

Other factors, like exercise fidelity, could also affect the association between compliance and injury. Improper exercise technique could trigger symptoms, especially if the player has an ongoing gradual onset knee injury like patellofemoral pain, which is a common condition in youth female athletes [13]. High exercise fidelity in combination with correct progression level will probably optimise the workload and increase the injury preventive effect [13, 15]. Coaches may be helped with practical advice on how to instruct athletes during implementation of the exercises, for instance using an external focus to improve technique [22].

### High vs. low compliance teams

In addition to a higher frequency of *Knee Control* use, coaches in the high team compliance group had adopted the full programme to a higher degree. Coaches in the high and low team compliance groups had generally similar opinions about prevention expectancies, which is in line with the previous research about predictors of successful implementation of an IPEP [18]. One noteworthy difference was that coaches in the high team compliance group believed that the players’ performance would increase from using *Knee Control*, while coaches in low compliance teams had the opposite perception. A recent study showed that IPEP adherence could be improved by promoting the performance enhancing effects [23], and our findings seem to support this. Other factors that could affect the uptake of the intervention are coaches’ task self-efficacy, i.e., how confident they are to use the IPEP in different situations [18], and if coaches think that injury prevention is a part of their role and responsibilities [23]. We have also previously reported that youth sport coaches desire support and confirmation from other peers, their club and sport associations, in using *Knee Control* [24]. Adherence may also be improved by providing greater adaptation possibility of an IPEP to suit the coaches’ and players’ needs and use of sport-specific equipment and skills training [23]. Our research group has therefore developed a *Knee Control*+ programme with more levels of progression, variation, and skills training, which has been pilot tested in football [20].

This study has some limitations. First, the coach and player surveys are not validated in their entirety but have sufficient face validity [18]. The consequence may be that the questions are interpreted differently. However, the questions are quite straight forward and should not leave much room for interpretation. Second, we acknowledge that post hoc dividing of teams and players into compliance groups may result in a skewed distribution of other injury risk factors such as previous injury between groups. Teams and players with historically high injury rates could be more eager to comply with *Knee Control*. It can also be noted that players in the high-dose group had an overall higher weekly floorball training and match exposure which can influence injury risk outcomes. Third, the team compliance allocation with an 80% cut-off was chosen arbitrarily based on the team compliance distribution with a distinct jump from 66 to 82% between two adjacent teams. A downside was a smaller sample (8 teams with 6 coaches) in the low team compliance group which makes data less robust. The more pragmatic categorization of player compliance groups was chosen to increase the feasibility of implementing the results with cut-offs on 1 and 2 *Knee Control* sessions per week. It is therefore difficult to directly compare the results with previous studies that have used a categorization based on population tertiles. Finally, a small number of players and injuries in some groups made sub-analyses less robust, and this was particularly evident for females where the risk of a type-2 error must be acknowledged.

Teams that had high compliance (high frequency of use) with *Knee Control* had also adopted the full programme to a greater extent, and since high player compliance was associated with a greater injury risk reduction, this should be stressed when educating coaches and athletes about injury prevention training. Players who had a *Knee Control* dose of minimum two sessions per week had the greatest effect, which means that coaches should implement the programme at all training sessions, or a minimum two times, and preferably three times, per week to ensure an effective player dose. Finally, promoting performance enhancing effects may improve IPEP adherence.

## Conclusion

Team compliance was high overall, with three out of four teams reporting use of *Knee Control* at > 80% of training sessions over the season. There were significant dose–response relationships between *Knee Control* dose and injury rate reductions in male floorball players but not in female players. Coaches should aim to use *Knee Control* on minimum three training sessions per week to reach an average player dose of 2 weekly *Knee Control* sessions. Teams with higher compliance showed a better utilisation fidelity with the programme. Coach demographics and baseline prevention expectancies were similar in team compliance groups, but coaches in high compliance teams perceived a more positive effect on player performance from using an injury prevention exercise programme.

## Supplementary Information

Below is the link to the electronic supplementary material.

Supplementary file1 (PDF 637 KB)

Supplementary file2 (PDF 195 KB)

Supplementary file3 (PDF 124 KB)

## Abbreviations

RCT: Randomised controlled trial
IPEP: Injury prevention exercise programme
ACL: Anterior cruciate ligament
OSTRC: Oslo Sports Trauma Research Center
SD: Standard deviation
IQR: Interquartile range
GEE: Generalized estimating equation
RR: Rate ratio
CI: Confidence interval
