# Supplementary material for: High compliance with the injury prevention exercise programme Knee Control is associated with a greater injury preventive effect in male, but not in female, youth floorball players
Source: Knee Surg Sports Traumatol Arthrosc. 2021 Jul 2;30(4):1480–90. doi: 10.1007/s00167-021-06644-2 (PMC9007760; doi:10.1007/s00167-021-06644-2)
Supplement: Supplementary file 1 — Supplementary file1 (PDF 637 KB) [file 167_2021_6644_MOESM1_ESM.pdf]

Additional file 1. Details of the running warm-up programme and *Knee Control* injury prevention exercise programme (IPEP) used in the intervention group

Exercise	Instructions	Sets repetitions/ duration
**Running exercises**	Course consists of 6-10 pairs of parallel cones, approximately 10 m wide and 20 m long. To be performed before each training session and match. Ensure good hip-knee-foot alignment during the running exercises	5 min
Running	Straight ahead	
Sideways jumps	With bent knees and low center of gravity	
Running	Slalom, with change of direction	
Sideways jumps	Jump with body contact; shoulder to shoulder	
Running	Alternate forwards and backwards running, with decelerations and accelerations	

**One legged knee squat**	**Slow movement with smooth turn, stable hip with horizontal pelvic position and non-supporting foot in front of the body with slightly flexed hip and knee. Knee flexion in alignment with the foot.**	
Level A	Hands on hips 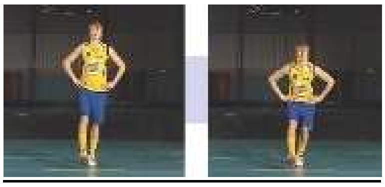	3×8-15 reps
Level B	Hold a stick 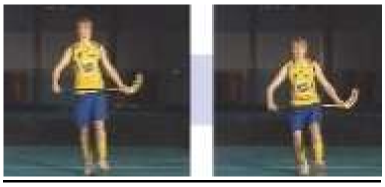	3×8-15 reps
Level C	Dribble in front of you 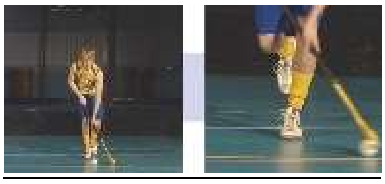	3×8-15 reps
Level D	Pass the ball against the court 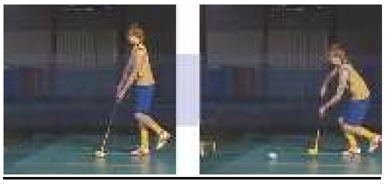	3×8-15 reps
Partner exercise	Both teammates stand on one leg with backs against each other. Hand over the floorball in different directions 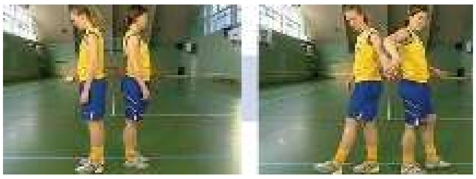	3×8-15 reps

**Pelvic lift**	**Supine position; lift the pelvis from ground to neutral position while keeping the back straight**	
Level A	Both feet on the ground and hands across the chest	3×8-15 reps
	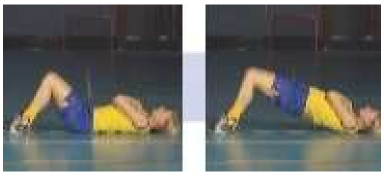	
Level B	Feet on a bench and hands across the chest	3×8-15 reps
	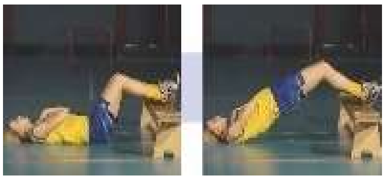	
Level C	One foot on the ground and the contralateral leg fixed straight, arms on the ground alongside the body. Lift the pelvis and abduct the straight leg	3×8-15 reps
	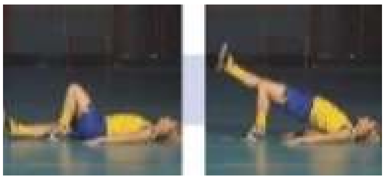	
Partner exercise	Supine position with feet towards each other. One foot on the ground and contralateral foot against the teammates foot	3×8-15 reps
	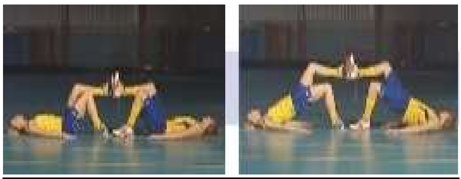	

**Two legged knee squat**	**Slow movement with smooth turn, back straight, and feet shoulder-width apart, pointing forwards and with soles in contact with the ground. The trunk should be upright.**	
Level A	Hands on hips	3×8-15 reps
	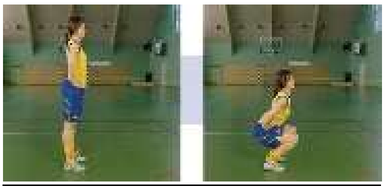	
Level B	Hold stick in front of the body with straight arms	3×8-15 reps
	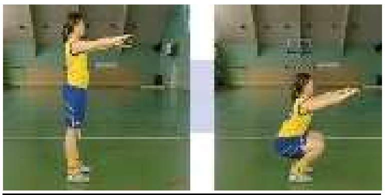	
Level C	Hold stick over the head with straight arms	3×8-15 reps
	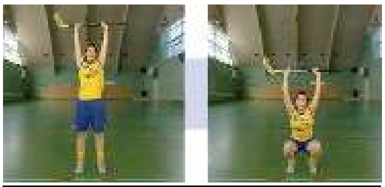	
Level D	Same as level C but continue movement and rise up on the toes after returning to the starting position and stay in the elevated position briefly	3×8-15 reps
	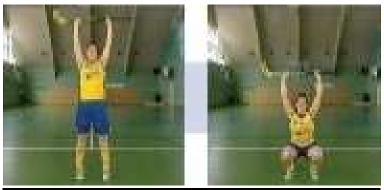	
Partner exercise	Teammate stands in front of you, facing towards you. Hold two sticks, one in each hand	3×8-15 reps
	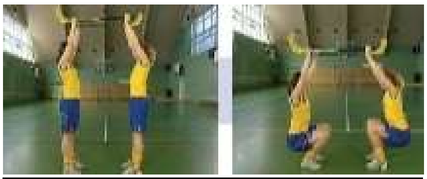	

**The bench**	**Lift body and keep it in a straight line. Elbows placed beneath the shoulders with 90° flexion of the shoulders and elbows**	
Level A	Prone position; support on knees and on lower arms with elbows kept beneath the shoulders 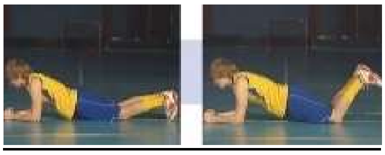	15-30 s
Level B	Same as level A but with support on the tip of the feet 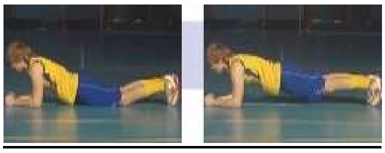	15-30 s
Level C	Same as level B, but raise one foot at a time up and down; alternate sides 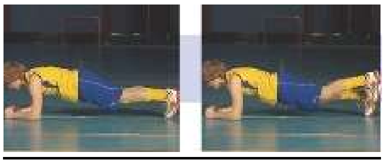	15-30 s
Level D	Lie sideways with support on the foot and lower arm with the elbow beneath the shoulder and the other hand on the hip; lift the hip off the ground, stay briefly in that position with good control, slowly return to the starting position. 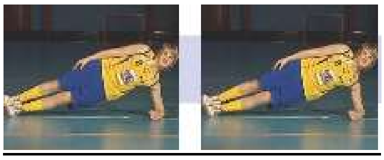	5-10 reps
Partner exercise	Same as level A but teammate face to face. Pass a floorball with the hand 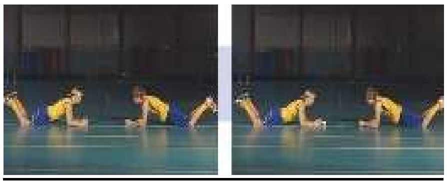	15-30 s

**The lunge**	**Take a deep step forwards with a marked knee lift and soft landing; the rear knee should not touch the ground. Keep good knee-over-foot alignment. Trunk control maintained with minimum lateral movement and stable hips. Foot placed pointing forwards**	
Level A	Hands on the hips; move forwards with each step	3×8-15 reps
	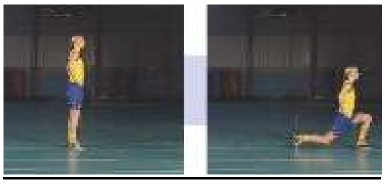	
Level B	Hold the stick in front of the body; move forwards with each step	3×8-15 reps
	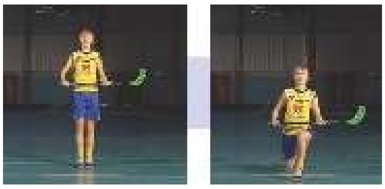	
Level C	Same as level B but continue the movement and rise up on the toes between each lunge	3×8-15 reps
	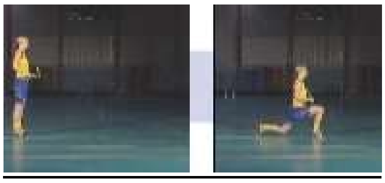	
Level D	Same as level B, rotate the upper body while stepping forwards and position the stick laterally to the front leg; move forwards with each step and alternate sides	3×8-15 reps
	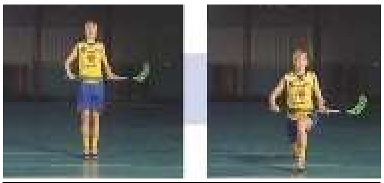	
Partner exercise	Teammate stands in front of you 5 m away; pass the floorball, perform a forward lunge, and receive a pass back	3×8-15 reps
	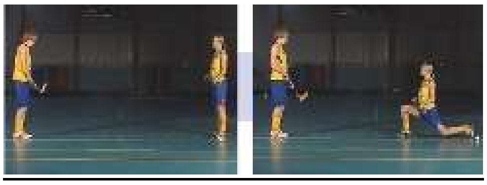	

**Jump/landing**	**Jump with a controlled and soft landing; stay briefly in the landing position. Keep good knee-over-foot alignment. Trunk control maintained with minimal motion. Knee control maintained with minimal wobble sideways.**	
Level A	Stand on one leg with the knee slightly bent and hands on the hips; make a short forward jump and land on the same foot; then jump backwards to the starting position 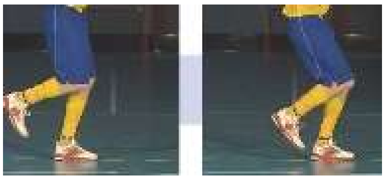	3×8-15 reps
Level B	Stand on both feet shoulder-width apart and hold a stick; make two forward jumps and land on one foot after the third jump with a controlled and soft landing; alternate sides 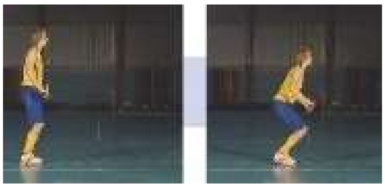	3×8-15 reps
Level C	Same as level B but rotate the body 90° before landing on one leg, alternate sides 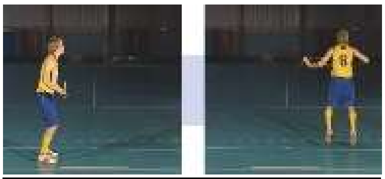	3×5 reps
Level D	Sideways jump on one foot and dribble with a ball and stick in front of you 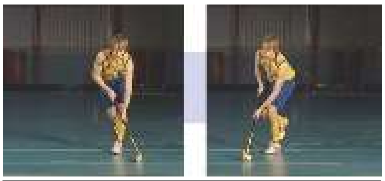	3×5 reps
Partner exercise	Teammate stands in front of you approximately 5 m away; make sideways jump on one foot and pass the floorball to your teammate. Jump back to the starting position and receive a pass back 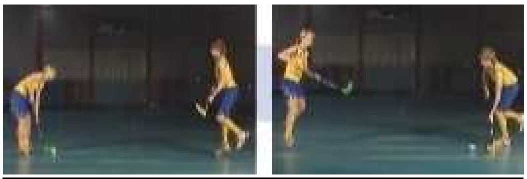	3×8-15 reps
