# Supplementary material for: High compliance with the injury prevention exercise programme Knee Control is associated with a greater injury preventive effect in male, but not in female, youth floorball players
Source: Knee Surg Sports Traumatol Arthrosc. 2021 Jul 2;30(4):1480–90. doi: 10.1007/s00167-021-06644-2 (PMC9007760; doi:10.1007/s00167-021-06644-2)
Supplement: Supplementary file 2 — Supplementary file2 (PDF 195 KB) [file 167_2021_6644_MOESM2_ESM.pdf]

Additional file 2. Coach surveys, selected parts relevant to this study

Baseline coach survey

Sex

Male

Female

How long have you been coaching a floorball team?
Since _____________ (year)

Questions about injury risks in floorball and the *Knee Control* Program

(circle the number, 1-7, that best matches your opinion)

What is your opinion about the overall injury risk in floorball?

	1	2	3	4	5	6	7	
Low	Extremely	Quite	Slightly	Neither	Sightly	Quite	Extremely	High

In general, how preventable do you think floorball injuries are?

	1	2	3	4	5	6	7	
Not preventable	Extremely	Quite	Slightly	Neither	Slightly	Quite	Extremely	Preventable

My knowledge about preventing injuries in floorball is…

	1	2	3	4	5	6	7	
Poor	Extremely	Quite	Slightly	Neither	Slightly	Quite	Extremely	Good

In your opinion, what would happen to a floorball player's overall risk of injury if he/she participated in injury prevention training?

	7	6	5	4	3	2	1	
Decrease	Extremely	Quite	Slightly	Neither	Slightly	Quite	Extremely	Increase

What do you think would happen to a floorball player's performance if he/she did injury prevention training regularly?

	1	2	3	4	5	6	7	
Decrease	Extremely	Quite	Slightly	Neither	Slightly	Quite	Extremely	Increase

Post season coach survey

When, during the floorball training, did you use the *Knee Control* programme?

- ☐ Before training session
- ☐ Beginning of training session
- ☐ During training session
- ☐ After training session
- ☐ It varied

How often did you use the different exercises in the programme? (tick the table, one answer per exercise)

	Never	Rarely	Mostly	Always
Running warm-up				
One legged knee squat				
Pelvic lift				
Two legged knee squat				
The bench				
The lunge				
Jump/landing				

Each main exercise is available in five different variants at different levels of difficulty. How did you use the programme at training?

- ☐ We used the same exercise variant at each training session
- ☐ We selected different exercise variants across the season for variation
- ☐ We have chosen more difficult exercise variants over time
- ☐ We adapted exercise variants for the individual player
- ☐ We did like this:

  ____

What exercise variants did you use most often? (mark with one or more crosses)

	Level A	Level B	Level C	Level D	Partner exercise
One legged knee squat					
Pelvic lift					
Two legged knee squat					
The bench					
The lunge					
Jump/landing					

How many rounds (sets) did you practice the same exercise? ______________________ rounds

How much time did you spend on the *Knee Control* programme during a training session?
_________min
