# Supplementary material for: High compliance with the injury prevention exercise programme Knee Control is associated with a greater injury preventive effect in male, but not in female, youth floorball players
Source: Knee Surg Sports Traumatol Arthrosc. 2021 Jul 2;30(4):1480–90. doi: 10.1007/s00167-021-06644-2 (PMC9007760; doi:10.1007/s00167-021-06644-2)
Supplement: Supplementary file 3 — Supplementary file3 (PDF 124 KB) [file 167_2021_6644_MOESM3_ESM.pdf]

Additional file 3. Coach reported use of the running warm-up and each *Knee Control* exercise in the high and low compliance groups, from post-season survey.

Knee Control exercise	Never or rarely n (%)	Mostly or always n (%)	Progression level[a]
Running warm-up			
- Low compliance	1 (25)	3 (75)	n.a.
- High compliance	1 (6)	16 (94)	n.a.
One legged knee squat			
- Low compliance	3 (75)	1 (25)	A
- High compliance	1 (6)	16 (94)	B
Pelvic lift			
- Low compliance	2 (50)	2 (50)	A
- High compliance	3 (18)	14 (82)	C
Two legged knee squat			
- Low compliance	2 (50)	2 (50)	A
- High compliance	0	17 (100)	C
The bench			
- Low compliance	1 (25)	3 (75)	A/B
- High compliance	1 (6)	16 (94)	B
The lunge			
- Low compliance	1 (25)	3 (75)	A/B
- High compliance	1 (6)	16 (94)	B
Jump/landing technique			
- Low compliance	3 (75)	1 (25)	A
- High compliance	3 (18)	14 (82)	B

High compliance (*n*=23) 80-100% of *Knee Control* use, low compliance (*n*=7)[b] <80% use

Response options were fixed: never/rarely/mostly/always

Most reported progression level among teams in each compliance group, A easiest – D most advanced

N.a. not applicable

[a]Missing for 2 teams in the high compliance group

[b]Missing for 1 team in the low compliance group
